# Online self-evaluation: the EFL writing skills in focus

**DOI:** 10.1186/s40862-022-00135-8

**Published:** 2022-05-01

**Authors:** Khaled Nasser Ali Al-Mwzaiji, Ali Abbas Falah Alzubi

**Affiliations:** English Department, Najran University, P.O. 1988, Najran, Saudi Arabia

**Keywords:** EFL writing, Online education, Self-evaluation strategy

## Abstract

Students need more independent roles to evaluate their learning performance, especially in the absence of teachers’ feedback due to some constraints such as technology and poor experience. This study investigates the potential of online self-evaluation on students’ writing skills in English as a foreign language (EFL). It examines the preparatory year (PY) level one students’ most improved EFL writing areas and perceptions when they use online self-evaluation in writing. In this quasi-experiment, two groups of PY EFL writing program students (N = 60) participated in the study for the first semester 2020–2021. A mixed-method design was applied: Self-monitoring checklists, self-evaluation questionnaires, achievement tests, and students’ portfolios. Findings revealed that most of the learners’ mistakes were in punctuation marks, capitalization, informal language, and subject-verb agreement. It was also perceived that learners were doing fine about where and how they should be. The study proved that the students’ self-evaluation and performance in writing correlated significantly; however, the effect size was low. Based on the research findings, it is recommended that training programs on strategy are to be boosted, especially in institutions where learners receive less feedback.

## Introduction

The outbreak of Covid-19 pandemic has transferred completely the educational process into a new phase (Zhu and Liu, 2020, cited in Kalaichelvi & Sankar 2021); and ‘many universities opted for an online mode of teaching’ (Nuraihan, Nor Shidrah, and Jantima 2020; Ajmal, et al. 2020); thus technology integration and the Internet in education have become inevitable tools nowadays. Thus, with the enlarging role of technology, some roles of the educational process parties must change, as education is becoming more student-centered: This approach emphasizes the learners’ engagement in all processes of learning like planning, setting goals, choice of materials, and assessment as learners are believed to be the first and last product of the whole process of education; and when they are incorporated in such processes, they will feel more responsible towards their learning and hence, their progress improves greatly. Self- evaluation, an indirect metacognitive language learning strategy, in EFL context is a significant issue, especially in online learning mode. Learners who consciously self-evaluate themselves can better recognize their mistakes and the reasons for committing them. Also, they can observe their EFL learning competence and check their progress. With the decreasing chances of immediate feedback by teachers in online learning mode due to technology hindrances and the learners’ need to compensate for this and seek maximum learning benefits, students will need to learn how to self-monitor and self-evaluate their EFL writing processes. This study is motivated by the significance of the topic for learners acquiring EFL writing skills and little research on examining self-evaluation strategy and its role in impacting their EFL writing skills in online mode. Therefore, the study is of significance through exploring learners’ most improved EFL writing areas and correlating the use of self-evaluation strategy and their EFL writing performance in the online learning mode.

## Literature review

Self-evaluation comes under indirect language learning strategies of metacognition that allow learners to control their language learning process (Oxford, 1990). EFL learners can make use of their mistakes to improve their learning strategies. Doing so requires that they recognize the mistakes they commit and have a chance to correct them.

Evaluation of foreign language learning involves two strategies: Self-monitoring and self-evaluation. Self-monitoring means the identification of ‘errors in understanding or producing the new language’. Self-evaluation refers to ‘evaluating one’s own progress in the new language’ (Oxford, 1990, p.140). Self-monitoring is the learners’ conscious decision to notice and correct their mistakes in EFL. In addition, learners can make use of their mistakes to identify why they commit these mistakes and try to avoid them in later stages. Learners can be provided with a checklist to monitor their errors in spelling, capitalization, punctuation, vocabulary, grammar, sentence/ paragraph structure, and/ or organization, and unity and cohesion. In self-evaluation, learners can rate their language learning competence through diaries, journals, or checklists. Sentence structure, written organization, accuracy, social appropriateness, and power of arguments can be the most aspects to be self-evaluated (Oxford, 1990). They can also review some samples of their own work, note the style and content, and evaluate their progress over time.

The learners’ use of self-evaluation strategy is less effective if not proceeded by training programs that help them be familiar with this aspect: Learners need to know what the strategy is, how to employ it, and practice as many samples as they can to help them be more competent and have a good command of mistake knowledge, so they are aware with and mature enough to self-evaluate their writing tasks, especially in online learning modes where the teacher’s immediate feedback may be less. Therefore, learners need to learn and employ high-order skills (metacognitive language learning strategies) like self-evaluation to claim more roles of responsibility and be in charge of their language learning (Alzubi, 2019; Alzubi & Singh, 2017; Alzubi et al., 2019).

### Previous research on self-assessment in writing in EFL context

Writing self-assessment has been extensively researched in conjunction with a number of some writing skills-related aspects in the EFL context such as academic improvement (Abolfazli & Sadeghi, 2012; Butler & Lee, 2010; Cömert & Kutlu, 2018; Elgadal, 2017; Fahimi & Rahimi, 2015; Heidarian, 2016; Sadeghi & Abolfazli, 2015; Thongpai & Deerajviset, 2017), ability to write (Butler & Lee, 2010), autonomy/ self-regulation (Barfield & Brown, 2007; Fathi et al., 2017; Ghadi, & Khodabakhshzadeh, 2016; Kostons et al., 2012), motivation (Heidarian, 2016), attitudes (Fahimi & Rahimi, 2015; Sadeghi & Abolfazli, 2015), accuracy (Butler & Lee, 2010; Purwanti, 2015; Rodliyah et al., 2017). Such aspects were found to have been affected by the EFL learners’ employment of self-assessment in writing assignments.

Previous research has found a positive correlation between students’ self-assessment and their academic achievement in EFL writing. Abolfazli and Sadeghi (2012) found that Iranian university students who used the self-assessment strategy outperformed students who used either peer-assessment or teacher-assessment in the achievement test of an EFL writing course. Also, Butler and Lee (2010) also reported that EFL Korean school learners who applied self-assessment tools improved their language learning performance and accuracy. However, this improvement was marginal due to the misunderstanding of self-assessment purposes and teachers’ provision of feedback. In addition, Sadeghi and Khonbi (2015) concluded that ESP Iranian undergraduates, who used self, peer, and teacher-assessment means scored better in the post test and had positive attitudes towards self-assessment.

Heidarian (2016) suggested that the use of self-assessment strategy through which they were able to find their mistakes and thus corrected them improved EFL Iranian learners’ writing performance. Thongpai and Deerajviset (2017) claimed that 68 EFL Thai undergraduates who learned using reflective learning styles scored higher in the posttest. Also, self-assessment checklists helped students monitor their weaknesses and strengths in writing, especially in the organization aspect. Nevertheless, they expressed their comfort with getting feedback from the teacher to self-assess their writing products. Finally, Cömert and Kutlu (2018) suggested that EFL Turkish undergraduates who used the self-assessment strategy fostered their learning of writing skills at the level of the paragraph, language use, and content.

In addition, self-assessment strategies had positive effects on some other writing-related aspects such as writing ability as Meihami and Varmaghani (2013) concluded that self-assessment would positively contribute to Iranian students’ essay writing abilities in an ESP writing course. Also, Fahimi and Rahimi (2015) were also of the view that raising EFL Iranian students’ knowledge about how to revise and assess their essay writing would foster their writing skills. In addition, EFL students’ attitudes towards self-assessment in writing had turned positive as reported by Purwanti (2015), who concluded that EFL Indonesian students had positive attitudes towards self-assessment of their essay writing, and they could self-evaluate their writings at the level of the phrase, lexical, content, surface, but failed to improve their grammar accuracy. Furthermore, Rodliyah et al. (2017) examined Indonesian university students’ use of self-correction in an academic writing course and found that although students were able to identify mistakes, they could not correct some of them.

Students’ use of self-assessment strategies has affected their motivation and cooperation in EFL writing skills. Heidarian (2016) recommended that the use of the self-assessment strategy by which they were able to find their mistakes and thus corrected them motivated them to learn English through having a more student-centered learning environment and increasing cooperation between students and teachers. Elgadal (2017) reported on EFL Libyan undergraduates’ implementation of self-assessment to revise their essay writing and showed that students made corrections at the level of surface and meaning. Also, the participants welcomed the incorporation of self-assessment strategy in the EFL writing course.

Various assessments such as self-assessment or peer-assessment could play a role in boosting EFL learners’ self-regulated learning processes. Fathi et al. (2017) revealed that Iranian university students who employed self-assessment or peer-assessment strategy improved their self-regulated learning skills in the EFL writing context. Also, Kostons et al. (2012) concluded that the Dutch secondary students had better chances to self-regulate their learning process and thus increase their amount of knowledge through the employment of self-assessment and self-material selection skills. In addition, Ghadi and Khodabakhshzadeh (2016) explored the effect of Iranian students’ use of electronic peer assessment on their EFL writing ability and autonomy and suggested that the learners’ autonomy was enhanced.

The existing literature on using self-assessment strategies has been addressed in correlation with learners’ EFL writing improvement level, motivation, and attitudes. However, there has been very limited research on applying self-evaluation strategies by tertiary students in Saudi Arabia. Also, few if not studies are there on examining self-evaluation strategies in online modes of education. Therefore, this study is significant as it investigates the impact of PY Saudi students’ implementation of self-evaluation strategies on their improvement level of EFL writing skills, progress, and areas. The study addressed the following questions (RQs):

- ***RQ1:*** *What are the EFL writing areas in which PY students have improved in the online learning mode?*
- ***RQ2:**** Is there any significant correlation between PY students’ self-evaluation strategy and their EFL writing performance in the online learning mode?*

## Methodology

The explanatory mixed methods research design is adopted in this study because of the need to explain the effects of online self-evaluation on the students' performance in the EFL writing skills. The design was applied to collect the data through self-monitoring checklists, a self-evaluation questionnaire, an achievement test, and students’ portfolios.

### Population and sample

The target population of the study used PY students at Najran University. PY is a program for two semesters in which high school students in the science track at secondary school study courses on English skills, computer skills, communication and ethics skills, and mathematics before they specialize in the medical, engineering, and computer science faculties. The study sample had two male groups (N = 60): Experimental (N = 30) and control (N = 30); and the participants were chosen purposefully to serve the study objectives. To some great extent, the two groups were homogenous in terms of nationality (Saudi), gender (male), age (average 19), level (one), educational background (high school\ science track), L1 (Arabic), L2 (English), English level in the placement test (pre-intermediate). While the experimental group studied writing skills using the self-evaluation strategy after having received the necessary training, the control group studied writing skills through the followed traditional ways of evaluation: teacher’s evaluation. Right before the experiment started, the participants were oriented about the study objectives and their roles in the study. Also, the participants who volunteered to take part in the study, completed two mailed consent form copies, kept one copy and returned the other to the researcher. The consent form included all the information about the study like the title, objectives, roles, risks, assurance of data confidentiality, and researcher’s personal details.

### Instruments and materials

The data were collected using the following instruments: A self-monitoring checklist, a self-evaluation questionnaire, tests, and students’ portfolios.

### Self-monitoring checklist

The self-monitoring checklist was developed based on the textbook topics. It aimed to help students recognize and correct their errors in writing tasks. It included issues that students should be aware of in social and academic writing, which are explained in the heading of the textbook. There were eight topics divided into two levels: Writing simple and compound sentences; a job, a classroom and writing paragraphs; a friendly letter, a postcard, a blog, a journal, a formal paragraph about a favorite topic. The eight-prepared checklists in the textbook were used to allow the participants in the experimental group to self-monitor their writing assignments after receiving the necessary training. The items in the self-monitoring checklist covered sentence structures, punctuation, capitalization, contractions, pronouns, types of sentences, use of conjunctions, forms of writing, and paragraph writing. In addition, academic writing covers issues like paragraph format and types of paragraphs.

### Self-evaluation questionnaire

The self-evaluation questionnaire was used to help students rate their level progress of writing based on a number of items adopted from Oxford (1990). The self-evaluation questionnaire consisted of three sections: personal information, understanding a student’s writing in English by people, peers, and teacher, improvements in terms of quality and quantity, and compensation for the loss of words in writing (N = 5 items), overall progress in writing (N = I item). The participants rated their responses to the self-evaluation questionnaire on a three-Likert scale. Items (1, 2, & 5) were rated using the following responses: Never, sometimes, and always. Items (3 & 4) were rated responses as follows: Little extent, moderate extent, large extent. The overall progress in writing was rated using: Doing just fine, about where I should be, not too bad, nothing to worry about, serious problems.

### Test

The final test of the writing skills course prepared by the department concerned was used to correlate the students’ conscious use of self-monitoring strategies with writing performance. The test was administered online via Blackboard. It was an objective question test that included multiple-choice, true/false, matching, fill in the blank, and jumbled sentence questions. The achievement test of the writing course had a total of 50 items on the topics covered in the syllabus breakup and an allotted time of two hours.

### Portfolios

Students’ portfolios included the participants’ completed essay assignments in the writing skill course. They were instructed to upload the assignments online. Two pages of Padlet website have been created and kept open for students a whole semester: One for the experimental group (https://padlet.com/aliyarmouk2004/lzk2yr35i4upsqtz), and another for the control group (https://padlet.com/aliyarmouk2004/ehsxfthn9qzcvya4). All the assignments were downloaded and kept in files. The collected data from portfolios were aimed to check and compare the areas of improvement in the writing skills between the participants in the experimental group and the control group.

### Materials

The textbook used in the study is the prescribed course at the PY program: Writing Power 1 by Karen Blanchard (2013). It has four parts: Language Use (Sentence Basics, Adding Information to Sentences, Simple and Compound Sentences), Social and Personal Writing (Friendly Letters, Emails and Blogs, Journals), Academic Writing (Paragraph Basics and Topic Sentences, Supporting and Concluding Sentences, Listing Paragraphs, Writing Instructions), and Vocabulary Building (Vocabulary Building Strategies, Dictionary Skills, Word Parts, How Words Work Together, Creative Writing). According to the syllabus of the 141 writing course, only the first three parts are being taught to the students; Part 4 is excluded. The EFL writing course (141 Writing) aims to enhance a number of objectives relating to the proposed research objectives that pertain to building students who can self-monitor and correct their mistakes and self-evaluate their progress. Each unit in the textbook is designed to assist students to brainstorm thoughts, select, organize, and develop ideas, draft a text, check and revise the text, and publish their work by sharing it with classmates and teacher. Due to the Covid-19 pandemic, the course is being taught online through Blackboard. Blackboard is an online educational platform at the university level and is used in most Gulf countries.

### Procedures for data collection

Data collection included a number of procedures. Students in the experimental group were oriented and trained on the use of self-monitoring strategies. The training course included two aspects: Theory and practice. Students were introduced to evaluation definitions, types, importance, strategies, and characteristics with a special emphasis on self-monitoring strategies. They also practiced self-monitoring using a checklist. After that, they studied the writing textbook applying the self-monitoring strategies. Students were instructed to do their writing assignments and use the self-monitoring checklists to check their errors and correct them. The students had two-hour sessions every week, and the treatment program lasted for one semester. Their writing assignments were collected in a portfolio and later analyzed in terms of frequencies compared to those students in the control group who learned without self-monitoring strategies. Two pages for the control group and the experimental group on Padlet were created for students to upload their assignments. At the end of the course, students who took the treatment were asked to rate their writing level using an evaluation questionnaire. The questionnaire was prepared via Google Forms, and the link was passed to the students in the last class. Finally, their final achievement test marks were correlated with the students’ marks in the control group to calculate the effect size of the program.

### Data analysis

The data collected by the self-evaluation questionnaire and the achievement test were analyzed using SPSS (23) in terms of descriptive and inferential statistics, while the data to be collected by students’ portfolios were analyzed in terms of frequencies. The self-evaluation questionnaire validity was checked using Pearson correlation, the reliability was checked by Cronbach’s Alpha. Also, correlation between the participants’ use of self-evaluation strategy and their EFL writing performance in the online learning mode was analyzed using Mann–Whitney U test to extract the ordinal means between the two groups’ scores.

### Validity and reliability

The self-evaluation questionnaire was adopted from Oxford (1990). It aimed to measure the areas where students felt more improved in terms of people, teacher, and peers’ understanding for their writing, writing quality and quantity, and compensations for the loss of words. The questionnaire has piloted a sample of 15 students who were later excluded from the main study. Pearson correlation coefficient was computed for the scale and items as shown in the following table.

Table 1 shows that the values of Pearson correlation coefficients at the levels of items and overall of the scale are statistically significant at 0.05 or 0.01. This indicates that the scale is valid to measure what has been developed for.

**Table 1:** Self-evaluation questionnaire’s Pearson correlation coefficient

N	Item	Pearson correlation	Overall
1	When you write in the English language outside the classroom, do people generally understand your meaning?	Pearson correlation	.748**
		Sig. (2-tailed)	.001
		N	15
2	When you write in the English language in the classroom, do students and teacher generally understand your meaning?	Pearson correlation	.815**
		Sig. (2-tailed)	.000
		N	15
3	Has your writing improved since the beginning of the semester in terms of quality?	Pearson correlation	.917**
		Sig. (2-tailed)	.000
		N	15
4	Has your writing improved since the beginning of the semester in terms of quantity?	Pearson correlation	.742**
		Sig. (2-tailed)	.002
		N	15
5	Do you find ways to express yourself in writing even if you do not know all the words?	Pearson correlation	.671**
		Sig. (2-tailed)	.006
		N	15
6	On the basis of these questions, give yourself a rating on writing: 1. Doing just fine, about where I should be; 2. Not too bad, nothing to worry about; 3. Serious problems	Pearson correlation	.619*
		Sig. (2-tailed)	.014
		N	15
–	Overall	Pearson correlation	1
		Sig. (2-tailed)	
		N	15

*Correlation is significant at the 0.05 level (2-tailed)

**Correlation is significant at the 0.01 level (2-tailed)

Also, the internal consistency of the scale was measured using Cronbach’s Alpha. Table 2 displays the overall reliability of the scale.

**Table 2:** Self-evaluation questionnaire’s reliability

Cronbach's Alpha	No. of items
.837	6

Table 2 shows that the self-evaluation reliability scored 0.837 which is considered an acceptably good rate.

## Results

- ***RQ1:*** *What are the EFL writing areas in which PY students have improved in the online learning mode?*

PY students’ improved areas in EFL writing skills were investigated through their portfolios that were collected during the treatment program and a self-evaluation questionnaire that was administered at the end of the program.

### Portfolios

Students in the experimental group were instructed to write their assignment first, then use a checklist to check and correct their mistakes before uploading it in Padlet. All completed assignments of the students in the control group and experimental groups were selected to be analyzed. Numbers were selected for checking and comparisons. The checking number of errors between the control group and experimental group was based on the items in the checklist.

Table 3 shows the number of topics, number and types of mistakes, and submitted assignments by the participants in the experimental group and control group. Thirty-six mistakes were checked in the eight self-monitoring checklists. The total number of assignments submitted by the participants in the experimental and control groups reached 115. The experimental group submitted 79 (69%) assignments, while the control group submitted 36 (31%) assignments. The number of submitted assignments compared to the experimental group was more than double of those assignments by the control group, out of 79 assignments, 34 (41%) mistakes were committed by the participants in the experimental group whereas the control group had 49 (59%) mistakes. One explanation may be that the participants in the experimental group were encouraged by the self-evaluation strategies to write and correct the assignment for themselves. Most of the students’ mistakes were concentrated on very basic issues such as punctuation marks, capitalization, informal language, and subject-verb agreement. The most serious mistakes were found in the use of punctuation; while the experimental group had 17 (39%) mistakes, the control group committed 27 (61%) mistakes (see Appendix 1 for a sample of the students’ use of punctuation).

**Table 3:** Coding sheet of the participants’ EFL writing portfolios

No	Topics	No	Types of mistakes	No of the submitted assignments	Exp. group	Ctrl. group
1	Write sentences about a job	1	Subject	30 (17 exp.: 13 ctrl.)	0	0
		2	Verb		0	2
		3	Begins with a capital letter		3	1
		4	Ends with a period		3	3
		5	Personal pronouns and possessive adjectives		0	1
2	Write sentences about your classroom using there is/ are	1	Subject	15 (11 exp.:4 ctrl.)	1	0
		2	Verb		0	0
		3	Begins with a capital letter		0	0
		4	Ends with a period		0	5
3	Write compound sentences about hobbies	1	Subject and verb	22 (16 exp.:6 ctrl.)	1	3
		2	Connecting word		0	0
		3	Comma before the connecting word		8	7
		4	Ends with a period		6	11
4	Writing a friendly letter	1	Has a heading	14 (10 exp.:4 ctrl.)	0	1
		2	Has a greeting		0	0
		3	Has a body		0	0
		4	Has a closing and signature		1	0
		5	Has correct punctuation and capitalization		2	3
		6	Has contractions		1	1
5	Writing a postcard	1	Has a date	11 (7 exp.:4 ctrl.)	0	1
		2	Has a complete address		1	1
		3	Has a closing and signature		1	0
		4	Uses informal language		0	2
6	Writing a blog	1	Every sentence begins with a capital letter	10 (7 exp.:3 ctrl.)	0	1
		2	Every sentence includes correct punctuation		0	0
		3	Every sentence uses contractions where possible		2	1
		4	Every sentence uses capital letters for names of people, days, months, cities, and states		1	3
7	Writing a journal	1	Has an address	10 (8 exp.:2 ctrl.)	0	0
		2	Has a picture/ drawing		0	0
		3	Has phrases		2	2
8	Writing a paragraph about a favorite topic	1	Has a topic with a clear main idea	3 (3 exp.: 0 ctrl.)	0	–
		2	Has a supporting sentence about the main idea		0	–
		3	Has a concluding sentence with words from the topic sentence		1	–
		4	Has only compete sentences		0	–
		5	Has correct paragraph format, punctuation, and capitalization		0	–
		6	Has a correct title		0	–
–	Total	36		115 (79 exp.: 36 ctrl.)	34	49
					83	

Then appeared the misuse of capital letters: Five mistakes were committed by the experimental group, whereas the control group had seven mistakes (see Appendix 2 for a sample of the students’ use of capitalization).

The use of contractions and abbreviations had five mistakes by the experimental group and six mistakes by the control group (see Appendix 3 for a sample of the students’ use of contraction).

The control group had more issues with the subject-verb agreement (five mistakes) compared with the experimental group who had only two mistakes. However, students in the experimental group scored more mistakes (five) in format issues, whereas the control group had three mistakes (see Appendix 4 for a sample of the students’ use of subject-verb agreement).

To sum up, there is a difference in the number of mistakes by the participants in the experimental group in comparison with the control group in favor of the experimental group after considering the number of submitted assignments by each group. This finding is attributed to the use of the self-monitoring checklist by the participants in the experimental group. There is also more evidence that the submitted assignments of the experimental group participants witnessed some correction and deletion processes which indicates the employment of their self-monitoring checklists to correct their mistakes (see Appendix 5 for a sample of the students’ correction and deletion).

One more significant observation about the advantages of using self-monitoring checklists is that the more the task was bigger, the more the participants in the control group forgot about avoiding some mistakes they learn before. The self-monitoring checklist was a reminder for the participants to avoid mistakes and a source of help to only focus on the new mission.

### Self-evaluation questionnaire

By the end of the treatment program, a self-evaluation questionnaire was administered to assess the responses of the experimental group on the EFL writing areas in which they have improved: People, teacher, and peers’ understanding for their writing, writing quality and quantity, and compensations for the loss of words. The results of the self-evaluation questionnaire analysis are interpreted based on the three-Likert scale as the following: 1–1.66 = low, 1.67– > 2.33 = medium, 2.34–3.00 = high. Table 4 shows the descriptive statistics of respondents’ answers: Means (M) and standard deviations (SD).

**Table 4:** Descriptive statistics of the self-evaluation questionnaire

Item	N	Mean	Std. Deviation	Level
When you write in the English language outside the classroom, do people generally understand your meaning?	30	2.33	.479	Medium
When you write in the English language in the classroom, do students and teacher generally understand your meaning?	30	2.73	.450	High
Has your writing improved since the beginning of the semester in terms of quality?	30	2.60	.498	High
Has your writing improved since the beginning of the semester in terms of quantity?	30	2.53	.507	High
Do you find ways to express yourself in writing even if you do not know all the words?	30	2.47	.507	High
On the basis of these questions, give yourself a rating on writing: 1. Doing just fine, about where I should be; 2. Not too bad, nothing to worry about; 3. Serious problems	30	2.47	.629	High
Overall	30	2.52	.338	High

Table 4 above shows that the participants’ level in EFL writing was greatly improved at the overall scale (M = 2.52, SD = 0.338). The improvement is also reflected on the scale items. The participants’ ability to successfully communicate a message through their writing to their peers and teacher received the highest score (M = 2.73, SD = 0.450). However, people’s understanding of their writing outside the classroom had a medium level (M = 2.33, SD = 0.479). It is concluded that the participants’ progress in EFL writing is high, i.e., they are doing just fine about where they should be (M = 2.47, SD = 0.629).

- ***RQ2:*** *Is there any significant correlation between PY students’ self-evaluation strategy and their EFL writing performance in the online learning mode?*

Students’ use of self-evaluative strategies was correlated with their performance in EFL writing. The equality of data distribution was tested using the Kolmogorov–Smirnov test as depicted in the following table.

Table 5 reveals that the participants’ scores in the experimental and control groups are not normally distributed; therefore, in order to show the differences between the students’ score means in the experimental and control groups, the Mann–Whitney U test was employed to extract the ordinal means between the two groups’ scores as shown in the following table (Table 6).

**Table 5:** Kolmogorov–Smirnov tests of normality between the experimental and control groups

Group	Tests of normality					
	Kolmogorov–Smirnov^a^			Shapiro–Wilk		
	Statistic	Df	Sig	Statistic	Df	Sig
Control group	.191	30	.007	.883	30	.003
Experimental group	.226	30	.000	.889	30	.005

**Table 6:** Mann–Whitney U test for the experimental and control groups’ scores in the achievement test

Group	N	Mean	SD	Mean rank	Sum of ranks	Mann–Whitney U	Asymp. Sig. (2-tailed)	Z	Effect size
Control group	30	41.60	6.631	25.08	752.50				
Experimental group	30	45.50	3.665	35.92	1077.50	287.500	.016	− 2.413-	0.31
Overall	60								

Table 6 shows that among PY level one students in the writing skills course taking the final exam (N = 60), there was a statistically significant difference between the control group (M = 41.60, SD = 6.631) and the experimental group (M = 45.50, SD = 3.665), t (60), p ≥ 0.05 in favor of the experimental group. Therefore, there are differences in the writing scores between the groups: control and experimental. According to Cohen’s effect size value (d = 0.09), this size effect is considered a low practical significance.

## Discussion

The current study examined the effect of using the self-evaluation strategy on PY students’ writing performance and skills in an EFL context. Three main findings have emerged.

Analyzing the participants’ portfolios has revealed that the majority of their mistakes were reported in the use of punctuation marks, capitalization, informal language, and subject-verb agreement. The participants in the experimental group in comparison with the control group reported fewer mistakes. This finding might have been attributed to the use of the self-monitoring checklist by the participants in the experimental group that was evident in their submitted assignments which witnessed some correction and deletion processes. One more notice about the advantages of using self-monitoring checklists is that the bigger the task was, the more the participants in the control group commit mistakes they had learned before. The self-monitoring checklists were a reminder for the participants to avoid mistakes and a sort of help to only focus on the new mission. Heidarian (2016) suggested that the self-evaluation strategy is useful because it helps learners consciously improve their writing skills through locating, correcting, and thus avoiding mistakes. Hence, the students who learned using the self-evaluation strategy were able to locate mistakes and correct them, but they were unable to find and correct them all. In the same context, Thongpai and Deerajviset’s (2017) study reported that the students who utilized self-assessment checklists were better at monitoring their strong and weak points, especially in the organization aspect.

Another major finding reported in this study was that the participants’ improvement level in EFL writing areas: people, teacher, and peers’ understanding for their writing, writing quality and quantity, and compensations for the loss of vocabulary have received high perceptions. The participants’ ability to successfully communicate a message through their writing to their peers and their teacher has received the greatest improvement, whereas people’s understanding of their writing outside the classroom was medium. However, it is concluded that the participants’ progress in EFL writing is high, i.e., they are doing just fine about where they should be. This finding concurs with that by Cömert and Kutlu (2018), who revealed fostered writing skills at paragraph, language use, and content levels among EFL learners who employed self-assessment.

Finally, there were statistically significant differences between the scores means of the control group and experimental group in favor of the experimental group; however, the effect size was small. This finding may be attributed to a number of reasons such as the online administration of tests that might have allowed for more cheating, the nature of tests that were all multiple-choice type, and teachers’ less experience in preparing question banks, and inefficiency of distractors and discrimination questions. All of these factors may have affected the effect size of using self-evaluative strategies on improving EFL writing performance among the participants in the current study. The finding on the correlation between the learners’ use of self-evaluation strategy and their academic performance in EFL writing skills in the online learning mode revealed in this current study is supported by previous research. To cite some examples, the study by Butler and Lee (2010) revealed improvements in the students’ language learning performance after utilizing self-assessment tools. Also, the finding intersects with that by Sadeghi and Khonbi (2015), in which ESP students who employed self, peer, and teacher-assessment had scored better. In addition, the students’ improvements in EFL writing performance are in agreement with that by Heidarian’s (2016), where students who used the self-assessment strategy were able to improve EFL learners’ writing performance.

## Conclusion

The current study has emphasized the use of self-evaluation strategy by learners in EFL writing context in learning management systems like Blackboard. The impact of self-evaluation strategy on the learners’ EFL writing performance and skills was examined. The learners’ most mistakes were reported in the use of punctuation marks, capitalization, informal language, and subject-verb agreement. Also, it was found that the learners were doing just fine about where they should be. Finally, their use of self-evaluation strategy and performance in EFL writing correlated significantly in spite of the fact that the effect size was small. The study implicates that there should be a high priority focus on implementing metacognitive language learning strategies like self-evaluation that help learners acquire more learning responsible roles amid the decreasing roles such as immediate feedback of teachers in online education modes. Therefore, the study recommends the implementation of learners’ independent enhancement training programs like self-evaluation to compensate for the absence of teachers’ roles in online education. The study is limited as it uses a small sample which reduces the chances of detecting generalization. Also, only male learners were included in the study due to the education-based segregation in Saudi Arabia. Future research may focus on investigating the gender factor in the use of self-evaluation among learners in the EFL writing context. Also, the results of the study on using self-evaluation strategy can be taken as a ground for further investigation in EFL other skills.

## Data Availability

Please contact corresponding author for data requests.

## Abbreviations

EFL: English as a foreign language
L1: A mother tongue
L2: A second language, a foreign language
PY: Preparatory year
